# Endothelial Function Increases after a 16-Week Diet and Exercise Intervention in Overweight and Obese Young Women

**DOI:** 10.1155/2014/327395

**Published:** 2014-03-20

**Authors:** Lisa M. Cotie, Andrea R. Josse, Stuart M. Phillips, Maureen J. MacDonald

**Affiliations:** Department of Kinesiology, McMaster University, 1280 Main Street West, Hamilton, ON, Canada L8S 4K1

## Abstract

Weight loss improves endothelial function in overweight individuals. The effects of weight loss through combined aerobic and resistance training and caloric restriction on in vivo vascular measures and blood markers associated with the regulation of endothelial function have not been comprehensively examined. Therefore, we investigated brachial artery endothelial function and potential regulatory blood markers in twenty overweight women (30.3 ± 2.0 years) who participated in 16 weeks of aerobic (5 d/wk) and resistance training (2 d/wk) (combined: ≥250 kcal/d) and caloric restriction (−500 kcal/d versus requirement). Resting brachial artery flow mediated dilation (FMD) and circulating endothelin-1 (ET-1) and interleukin-6 (IL-6) were assessed at baseline and following the intervention. Relative and absolute FMD increased (before: 4.0 ± 0.5% versus after: 6.9 ± 0.6%, *P* < 0.05, and before: 0.14 ± 0.02 mm versus after: 0.23 ± 0.02 mm, *P* < 0.05, resp.), while body mass decreased (before: 86.9 ± 2.4 kg versus after: 81.1 ± 2.4 kg, *P* < 0.05) following the intervention. There were no changes in either blood marker (IL-6: before: 1.5 ± 0.2 pg/mL versus after: 1.5 ± 0.1 pg/mL, *P* > 0.05, and ET-1: before: 0.55 ± 0.05 pg/mL versus after: 0.59 ± 0.09 pg/mL, *P* > 0.05). 16 weeks of combined aerobic/resistance training and diet-induced weight loss improved endothelial function in overweight and obese young women, but this increase was not associated with changes in blood markers of vasoconstriction or inflammation.

## 1. Introduction

The vascular endothelium plays many roles, including contributing to the regulation of vascular smooth muscle tone. Endothelial dilatory capacity is commonly investigated noninvasively using the flow-mediated dilation (FMD) test. FMD measured in the brachial artery has been documented to correlate with pharmacological evaluations of coronary artery endothelial function [1]. Endothelial dysfunction may represent an early subclinical event in the development and progression of atherogenesis and is a known independent indicator of cardiovascular disease (CVD) [2, 3]. Aerobic exercise training improves endothelial function [4] and studies have reported a positive relationship between exercise-induced weight loss and endothelial function in overweight and obese men and women with established coronary heart disease [5]. A positive relationship between diet-induced weight loss and endothelial function in overweight and obese women with increased cardiovascular risk has also been reported [6–8]. No data exists on the effects of combined diet- and exercise-induced (aerobic and resistance) weight loss on FMD in otherwise healthy overweight and obese women.

At the molecular level, endothelial regulatory substances such as endothelin-1, a potent vasoconstrictor, and interleukin-6 (IL-6), a proinflammatory cytokine, have been measured in a number of exercise training studies [9–11]. Oxidative stress and inflammation are increased in overweight and obesity [12, 13]. As a result of increased oxidative stress, vascular inflammation also develops with obesity, as indicated by increases in expression of proinflammatory cytokines [12, 13]. Changes in endothelial function could be a key mechanistic link in the previously observed association between CVD and inflammation, as chronic inflammation has also been linked to endothelial dysfunction [9]. Specifically, proinflammatory cytokines may induce vasoconstriction by causing increased synthesis of endothelin-1 (ET-1) [9]. Although endothelial dysfunction is thought to be associated with a reduction in nitric oxide (NO), it has not been determined if increased ET-1 is a major contributor to this process. Increased ET-1 may contribute to the reduction of NO bioavailability, which is often observed with endothelial dysfunction [10].

IL-6 has been linked to various pathological states, including obesity [14–16], and is secreted from a variety of different cells, including vascular endothelial cells [17]. IL-6 is a mediator of the acute inflammatory response and contributes to chronic inflammation in obesity [18, 19]. Elevations in IL-6 are thought to stimulate the synthesis of ET-1 in the vasculature [9]. Approximately 33% of total IL-6 originates from adipose tissue [12] and it may play a key role in the relationship between adiposity, inflammation, and CVD. Importantly, an inverse correlation between IL-6 concentrations and endothelial function has been observed [11].

Aerobic exercise training leads to improved arterial health in many populations [20] and is involved in cardiovascular risk reduction. Research examining the effects of resistance training on arterial structure and function has, however, yielded conflicting results [21, 22] and the effects of combined aerobic and resistance training on arterial structure and function have not been comprehensively examined [23, 24].

The purpose of this study was to investigate the effects of a 16-week combined aerobic and resistance training program and hypocaloric diet on endothelial function, assessed with brachial FMD and circulating ET-1 and IL-6 in otherwise healthy overweight and obese young women. We hypothesized that brachial FMD would increase and that this would be inversely related to changes in circulating ET-1 and IL-6, suggesting these circulating markers may act as alternative indices of vascular endothelial function.

## 2. Methods

### 2.1. Participants

This study was part of a larger lifestyle intervention study: Improving Diet, Exercise, and Lifestyle (I.D.E.A.L) for Women study, which involved 90 participants [25]. A small subset of participants (*n* = 20) from the larger lifestyle intervention study volunteered to participate in the cardiovascular measurements for the current study, based on participant availability and consent. The Research Ethics Board of Hamilton Health Sciences approved the study. Twenty young, overweight female subjects with an average age of 30.3 ± 2.0 years (mean ± SE) participated in this study. Participants were otherwise healthy, overweight, or obese women (BMI: 32.4 ± 0.8 kg/m^2^). Other general inclusion criteria were sedentary lifestyle, regular menstrual cycle, and no vitamin or mineral supplementation. Participants were deemed healthy and thus eligible to participate based on their responses to a short medical screening questionnaire and measurement of serum lipids, glucose, and insulin concentrations all of which were normal (data not shown). All participants provided written informed consent before participating in the study.

### 2.2. Intervention

#### 2.2.1. Diet

The targeted total daily energy reduction throughout the study was −750 kcal/d (500 kcal/d by diet and 250 kcal/d through exercise). All participants received individualized diet counseling by study dieticians and research nutritionists on a biweekly basis. Every 2 weeks, participants provided a 3-day food record to track compliance with the intervention [25].

#### 2.2.2. Exercise Training

Participants completed 16 weeks of combined aerobic and resistance training as part of a targeted body composition-changing protocol. Participants exercised at the main fitness center at McMaster University. They engaged in various modes of aerobic exercise (stationary cycling, jogging on a treadmill, and walking on an indoor track) 5 d/wk and resistance exercise 2 d/wk with supervision. Each exercise session was designed to result in a minimum expenditure of 250 kcal in an effort to create a realistic workout routine for this population. During the week (Monday–Friday), subjects reported to the study office and were given a SenseWear Pro (BodyMedia, Pittsburgh, PA, US) arm band device to track energy expenditure [26]. Participants were requested to wear the SenseWear Pro device at home on several occasions randomly throughout the study in order to assess compliance with weekend workouts. The aerobic and resistance training programs have been described previously [25]. Briefly, participants engaged in a 2 d/wk resistance training protocol (upper body, lower body split). Weight progressions were made once the participants were able to successfully complete 3 sets of 10 repetitions at a given weight.

### 2.3. Cardiovascular Measurements

Testing sessions began with 10 minutes of supine rest to ensure representative resting measurements prior to the commencement of the vascular assessment. Continuous measurements of heart rate* via* single lead electrocardiograph (ECG) (model ML 123, ADInstruments Inc., Colorado Springs, Colorado, USA) and brachial blood pressure (BP) measurements* via* an automated applanation tonometer with oscillometric cuff calibration (model CBM-7000, Colin Medical Instruments, San Antonio, TX) were made. A FMD test was conducted to assess brachial artery endothelium-dependent function on the basis of previously established guidelines [27, 28]. All analogue signals (including those described below) were converted to digital by fast Fourier transform and sampled simultaneously at a sampling rate of 200 Hz using a commercially available data acquisition system (Power lab model ML 795, ADInstruments, Colorado Springs, Colorado, USA) and software program (LabChart 7.0, ADInstruments Inc., Colorado Springs, Colorado, USA).

### 2.4. Assessment of Flow-Mediated Dilation

FMD was assessed as previously described [29]. Briefly, with the participant in the supine position, the right arm was positioned and stabilized so that an optimal image of the brachial artery could be obtained in a comfortable position. An inflatable cuff was placed on the forearm, below the medial epicondyle [30], and remained deflated while baseline data were collected. Longitudinal B-mode ultrasound images of the left brachial artery were collected for five cardiac cycles using a 10-MHz linear array probe (System FiVe, GE Medical Systems, Horten, Norway) positioned 3–5 cm proximal to the antecubital fossa at a frame rate of 11 frames/second. Following acquisition of the B-mode image 30 seconds of continuous blood velocity in the brachial artery were acquired using pulsed-wave mode Doppler at a frequency of 4 MHz with the sample volume width set to insonate the entire artery. The forward and reverse audio signals from the pulse wave mode Doppler spectrum were processed by an external spectral analysis system (Neurovision 500 M, Multigon Industries, Yonkers, NY) and an intensity-weighted calculated mean signal was acquired (Powerlab model ML 795). This system applies a fast Fourier transformation to the raw audio signals to determine continuous intensity weighted mean blood velocity (MBV). The MBV was sampled at 200 Hz during the FMD tests using commercially available hardware (Powerlab model ML 795, ADInstruments).

To create the flow stimulus, a forearm cuff was instantaneously inflated, using a rapid cuff inflator (model E20 and AG101, Hokanson, Bellevue, WA) to a standardized, supra-systolic pressure of 200 mmHg to ensure arterial inflow occlusion and ischemia of downstream vessels and tissue [27]. The cuff was instantaneously deflated after 5 min. of occlusion and during the first 90 s after cuff release reactive hyperemic intensity weighted mean blood velocity signals were obtained as described above, followed by a B-mode image of the left brachial artery for 12 cardiac cycles at a frame rate of 11 frames/second. B-mode images were stored in Digital Imaging and Communications in Medicine (DICOM) format for later offline editing and analysis.

## 3. Data Analysis

### 3.1. Brachial Artery Blood Velocity

Preocclusion and postocclusion MBV were analyzed offline using LabChart 7 Pro for Windows (Powerlab ML 795, ADInstruments) in 3 s average time bins after correcting for angle of insonation (all ≤68°). Mean blood flow (preocclusion) was determined by multiplying brachial artery cross-sectional area by MBV. Recently published FMD guidelines advocate the normalization of FMD responses to the entire reactive hyperemic stimulus rather than normalizing to the peak stimulus [28]. The following equation was used to calculate shear rate (SR) for each participant for each 3 s bin [31]:

(1) $$\begin{array}{c} \text{Shear}\;\text{Rate}=8\times \left(\frac{\text{Velocity}}{\text{Diameter}}\right), \end{array}$$

where velocity represents the mean of the velocity profile in 3 s bins for the first 90 s postcuff release and the baseline brachial diameter (mm) is used for the artery diameter value. The area under the curve of the shear rate was calculated from the mean of the first point, using the trapezoid rule to obtain the area under the entire curve (GraphPad Prism version 4.00 for Windows, GraphPad Software, San Diego, California, USA). The entire reactive hyperemic stimulus was quantified as the shear rate area under the curve (AUC).

### 3.2. Brachial Artery Diameter

Using commercially available software (SanteDICOM Editor, Version 3.0.12, Santesoft, Athens, Greece), the end-diastolic frames, determined by the R-spike of the ECG trace, were extracted and stacked in a new DICOM file for determination of brachial artery diameters. A semiautomated edge detection software program (Artery Measurement System, Image and Data Analysis, Tomas Gustavsson, gustav@alumni.chalmers.se) was used to detect the vessel diameters within a specific region of interest. The program identifies the borders of the arterial wall within a selected region of interest on the basis of the contrasting intensity of brightness between the arterial wall and lumen, and it determines the diameter from approximately 100 points of measurement within the region of interest. Preocclusion diameters were determined from the average of the five end-diastolic frames. Peak postocclusion FMD diameter was determined from the 12 end-diastolic frames. From this data, the absolute FMD (mm) and relative FMD (%FMD) were calculated as follows [27]:

(2) Absolute  FMD=Peak  Diameter  (mm) −Baseline  Diameter  (mm)Relative  FMD=(Absolute  FMDBaseline  Diameter)×100%.

Relative FMD (%FMD) was normalized to the area under the entire SR curve and reported as

(3) $$\begin{array}{c} \text{Normalized}\;\text{FMD}=\left(\frac{\%\text{FMD}}{{\text{SR}}_{\text{AUC}}}\right). \end{array}$$

### 3.3. Blood Analysis

Overnight fasted venous blood samples were collected prior to and after 16 weeks of the intervention for further analysis. Serum was stored at −20°C for further analysis. Serum samples were analyzed using ELISAs for concentrations of interleukin-6 (R&D Systems Quantikine, Minneapolis, MN) and endothelin-1 (Enzo, Life Sciences Assay Designs, Farmingdale, NY) using the corresponding immunoassay kits.

### 3.4. Statistics

Results are presented as mean ± SE and differences were considered significant at *P* < 0.05. Data was analyzed using SPSS (Version 20). Mean differences were analyzed using Student's *t*-tests. Pearson correlations were used to assess relationships between flow-mediated dilation measures and blood markers.

## 4. Results

### 4.1. Participants

A total of twenty (*n* = 20) young healthy women (age = 30.3 ± 2.0 years; height = 163 ± 1 cm) completed the laboratory vascular testing. Body mass and BMI decreased after the intervention (Table 1).

### 4.2. Vascular Measures

There was no change in resting heart rate or resting mean arterial pressure (MAP), systolic blood pressure (SBP), or diastolic blood pressure (DBP) over time following the intervention (Table 2).

### 4.3. Arterial Function

**Figure 1:**
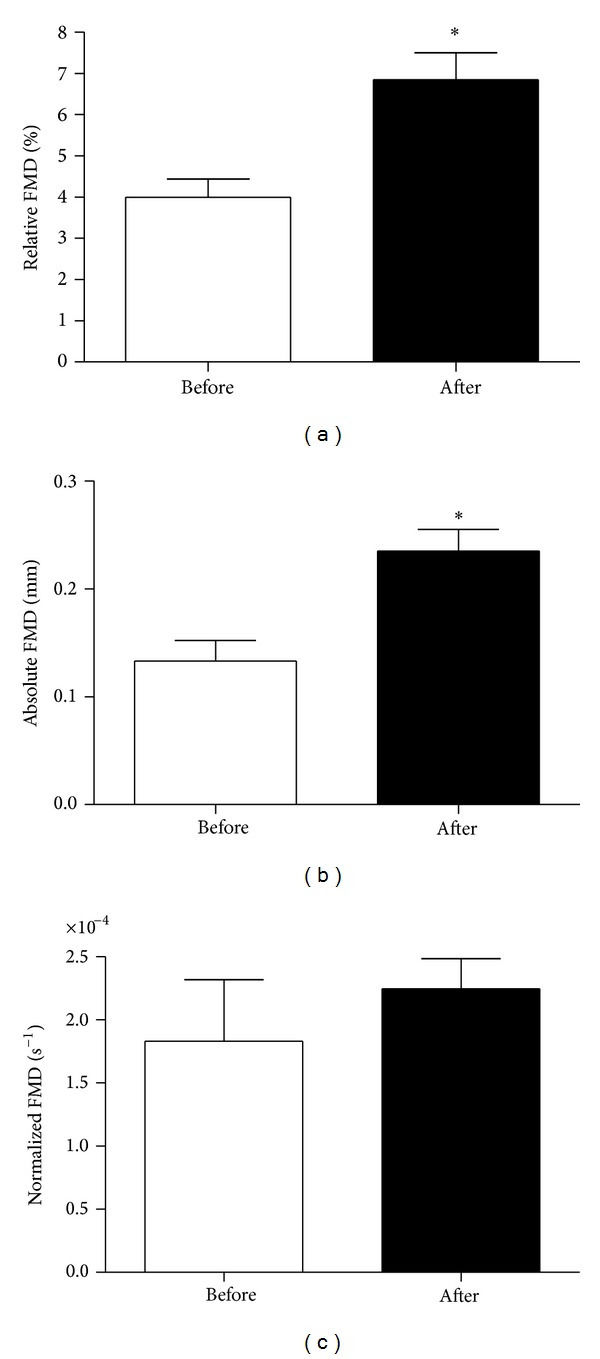
Changes in FMD from Week 0 to Week 16.

Relative (Figure 1(a)) and absolute FMD (Figure 1(b)) increased after the 16-week diet and exercise intervention. Data was pooled to represent the entire population before and after the intervention (relative: before: 4.0 ± 0.5% versus after: 6.9 ± 0.6%, *P* = 0.005; absolute: before: 0.14 ± 0.02 mm versus after: 0.23 ± 0.02 mm, *P* = 0.004). However, when FMD was normalized to shear rate, it was unchanged after 16 weeks (normalized: before: 1.8 × 10^4^ ± 4.8 × 10^5^ versus after: 2.2 × 10^4^ ± 2.3 × 10^5^, *P* = 0.45; Figure 1(c)). There were no relationships observed between weight loss (difference in body mass before and after) and increased FMD (relative, absolute, or normalized) following the intervention (weight loss versus change in absolute FMD: *r* = −0.118, *P* = 0.620; weight loss versus change in relative FMD: *r* = −0.070, *P* = 0.768; weight loss versus change in normalized FMD: *r* = −0.172, *P* = 0.468).

### 4.4. Arterial Structure

There was no change in resting brachial artery diameter after the 16-week diet and exercise intervention (Table 2).

### 4.5. Blood Markers

**Figure 2:**
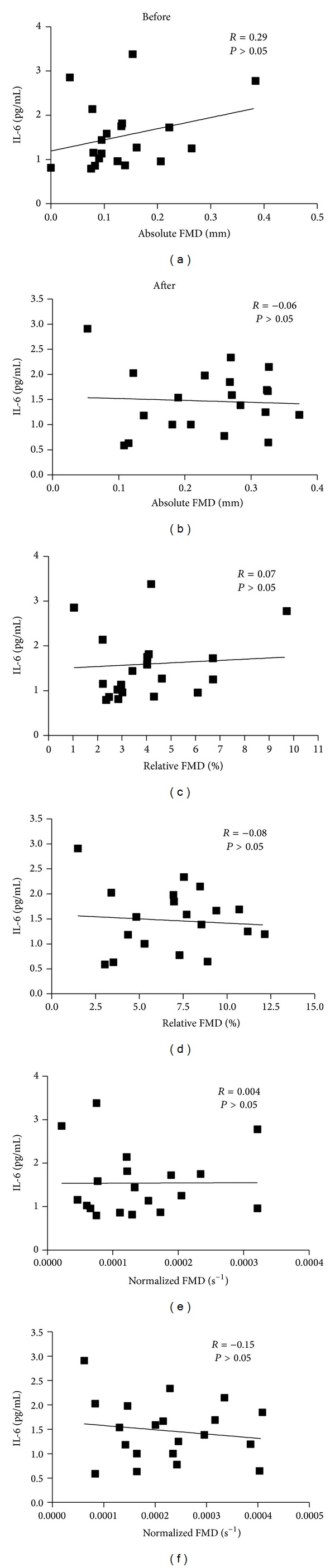
Relationships between FMD and IL-6 at Week 0 and Week 16.

**Figure 3:**
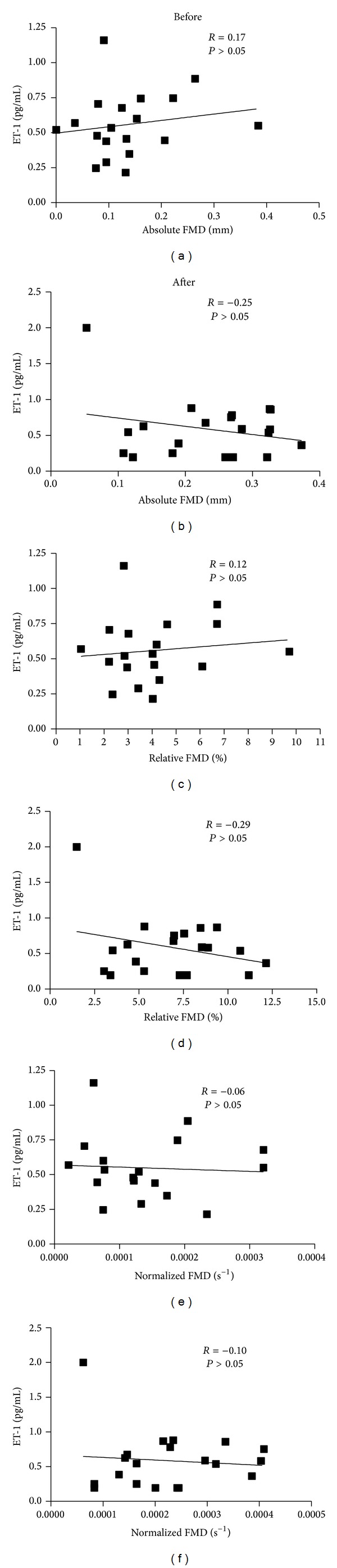
Relationships between FMD and ET-1 at Week 0 and Week 16.

There was no change in either of the serum markers (IL-6: before = 1.5 ± 0.2 pg/mL and after = 1.5 ± 0.1 pg/mL, *P* = 0.58, and ET-1: before = 0.55 ± 0.05 pg/mL and after = 0.59 ± 0.09 pg/mL, *P* = 0.73; *N* = 19, as one participant had undetectable ET-1 values). There were no relationships observed between any measures of FMD and IL-6 (Figure 2) or ET-1 (*N* = 19) before or after the intervention (Figure 3). One participant was removed from this analysis, as her ET-1 concentrations were undetectable.

## 5. Discussion

The main findings of this study were that brachial artery endothelial function assessed by relative (Δ%) and absolute (Δmm) FMD improved after the 16-week diet and exercise intervention; however, circulating serum concentrations of IL-6 and ET-1 were unchanged in our population of overweight women. Given the importance of endothelial function as an independent risk factor for CVD [2, 3] we view our findings as a relevant demonstration of what a multifaceted exercise- and diet-based weight loss intervention can do to alleviate CVD risk. The women included in this study were by definition sedentary [32, 33] which may have contributed to a reduction in baseline endothelial function. The exercise component of our intervention was therefore likely responsible, at least in part, for the improved endothelial function we observed.

Interestingly, while all of our subjects lost weight, the weight loss was not associated with changes in FMD or with reductions in markers of inflammation and so it is difficult to directly ascribe the changes in FMD to changes in body weight or indirectly through the influence this might have had on inflammation. Weight loss, with either diet [7, 8] and/or aerobic exercise [5] interventions, has previously been linked with increased endothelial function as measured by brachial FMD in overweight and obese women. While we did observe an improvement in FMD over time, there was no significant relationship between weight loss and improved endothelial function via brachial FMD measures with our combined diet and exercise intervention. Changes in FMD (%) with diet-induced weight-loss have been previously demonstrated; however, the reported differences are larger in magnitude than those observed in the current study. Mavri et al. (2011) observed an increase from 7.7% to 12.4% after 5 months of a low calorie diet in middle-aged obese women, whereas we observed an increase in FMD of 4.0% to 6.9% after 4 months. We do not singularly attribute the changes in FMD to the weight loss of the individuals or the dietary component of the intervention but rather to the combined vascular stimulus provided by the exercise and dietary intervention.

Aerobic exercise training has been shown to improve endothelial function in a variety of populations [34] including overweight postmenopausal women [22, 35]. We found a significant increase in FMD in overweight premenopausal women who performed combined aerobic and resistance training for 16 weeks. Our findings agree with those of Kwon and colleagues [22] who reported increases in relative FMD after a 12-week aerobic training program in overweight postmenopausal women (*P* = 0.032). Other studies have also shown increases in endothelial function as assessed by FMD using combined aerobic and resistance training interventions in other populations including young healthy men and men and women with type 2 diabetes mellitus [23, 24]. Our % change values are comparable to those found in the existing literature after combined aerobic and resistance training, suggesting the vascular stimulus from the exercise program may be the dominant mechanism associated with our observed increase in FMD. To our knowledge there are no studies to date that have investigated the effects of a combined diet and aerobic and resistance program on FMD in young overweight women and further research is needed to delineate the mechanisms associated with these changes.

Mechanistically, ET-1 and IL-6 are potential regulators of endothelial function. Inflammation has the ability to impair FMD as cytokines which may lead to increased vasoconstriction [9]. IL-6 contributes to chronic inflammation in conditions such as obesity and studies have identified elevated IL-6 levels in obese individuals [18, 19]. Chronic inflammation has been linked to endothelial dysfunction [9], and thus a decrease in endothelial dysfunction may be related to changes in inflammatory markers. Inverse relationships (*r* = −0.123, *P* < 0.0001) have been reported between serum IL-6 concentration and endothelial function [11]. ET-1 can decrease NO bioavailability either by decreasing its production or by increasing its degradation [10]. However, in the current study, ET-1 and IL-6 were unchanged following the 16-week intervention and there was no relationship observed between IL-6 or ET-1 and any of the FMD measurements suggesting that in our subject population IL-6 and ET-1 are not sensitive markers for changes in endothelial function observed during this type of lifestyle intervention.

Another potential explanation for our observation of no change in IL-6 in conjunction with the observed increase in FMD is the inclusion of resistance training in the exercise regime for our participants. There are conflicting results in the literature with respect to the effects of resistance training on circulating markers of inflammation. A recent study by Patterson et al. demonstrated that IL-6 levels were elevated following resistance training in older men [36], while Phillips et al. observed no change in IL-6 after resistance training in postmenopausal women [37]. It is also possible that the women in this study did not have chronic inflammation and thus we are observing a “floor” effect where even an intervention involving weight loss and exercise would not result in decreases in markers of inflammation.

It appears that ET-1 generally decreases following aerobic exercise training [38–40]. A recent study showed that ET-1 is reduced after just three weeks of aerobic training in middle-aged obese type 2 diabetic men and women [38]. Kasmay and colleagues (2010) observed a decrease in ET-1 when aerobic exercise training was accompanied with a low-calorie diet in patients with impaired glucose tolerance [39]. Maeda and colleagues (2003) observed a reduction in plasma ET-1 concentrations in older healthy women after 3 months of aerobic training [40]. In contrast we observed no differences in ET-1 concentration following 16 weeks of our exercise and diet intervention. While the populations involved in these studies were different than the current study, all were overweight and some were middle-aged women, thereby facilitating comparisons. It is possible the resistance-training component in our intervention attenuated any aerobic exercise training stimulated decreases in ET-1. Very little information exists regarding the effects of resistance training on ET-1; however, Maeda et al. (2004) observed a decrease in plasma ET-1 in young healthy men after 8 weeks of resistance training [41]. In this study we found no change in ET-1 after resistance training. More studies are necessary to better understand the relationship between ET-1 and resistance exercise training.

It is important to address the time course of changes in vascular structure and function with exercise interventions. It has been suggested that short-term (2–6 weeks) exercise training enhances eNOS and NO activity [42–46] while long-term (>8 weeks) training induces structural changes [46–48], marked by an increase in resting arterial diameter. This arterial remodeling is thought to normalize shear rate levels and thus may result in NO-mediated endothelial function returning to pretraining levels [34, 49, 50]. Original animal studies by Laughlin et al. [49, 50] and a compilation of data from different populations [46, 51–56] support this proposed time course of vascular function (peak at 6 weeks) and structure (>8 weeks) changes. In our study, FMD was still elevated at the 16-week time point, while resting brachial diameter was unchanged indicating that the time course of vascular changes with exercise may be different in overweight and obese young women and may also be influenced by the details of the intervention, including exercise mode, duration, intensity, and dietary modifications.

In conclusion, we observed an increase in endothelial function as measured by absolute and relative FMD following 16 weeks of a diet and exercise intervention that resulted in weight loss in overweight and obese premenopausal women. No changes were observed in blood markers, IL-6 and ET-1, which are often proposed as being mechanistically relevant in inflammation and vasoconstriction. Our study demonstrates that endothelial function is improved with weight loss and combined aerobic and resistance exercise and may be helpful for designing lifestyle interventions for overweight individuals at elevated CVD risk. Further research is warranted to better understand the mechanisms responsible for the observed changes.

## Figures and Tables

**Table 1:** Body composition measures before and following the 16-week intervention.

	Before (mean ± SE)	After (mean ± SE)	*P* value
Weight (kg)	86.8 ± 2.4	80.6 ± 2.4	<0.001
BMI (kg/m^2^)	32.4 ± 0.8	30.1 ± 0.7	<0.001

**Table 2:** Resting vascular measures before and following the 16-week intervention.

Resting variable	Before (mean ± SE)	After (mean ± SE)
Brachial diameter (mm)	3.39 ± 0.08	3.48 ± 0.09
HR (bpm)	65 ± 1	62 ± 1
MAP (mmHg)	81 ± 2	81 ± 3
SBP (mmHg)	116 ± 2	114 ± 3
DBP (mmHg)	63 ± 2	63 ± 13
